# Statin-Mediated Modulation of Nrf2 Signaling: Mechanisms and Therapeutic Implications in Atherosclerosis

**DOI:** 10.31083/RCM46903

**Published:** 2026-02-10

**Authors:** Basheer Abdullah Marzoog, Philipp Kopylov

**Affiliations:** ^1^World-Class Research Center “Digital Biodesign and Personalized Healthcare”, I.M. Sechenov First Moscow State Medical University (Sechenov University), 119991 Moscow, Russia

**Keywords:** statin, transcription factor, Nrf2, dyslipidemia, ischemic heart disease, atherosclerosis

## Abstract

Statins are the cornerstone of lipid-lowering therapy and exert significant pleiotropic effects, including antioxidant and anti-inflammatory actions, which contribute to statin-mediated cardiovascular benefits. A key mechanism underlying these effects is the indirect activation of the nuclear factor erythroid 2-related factor 2 (Nrf2) transcription factor. This review critically assesses the molecular pathways through which statins modulate Nrf2 signaling, primarily through the PI3K/Akt and ERK pathways, which results in the nuclear translocation of Nrf2 and the transactivation of a battery of cytoprotective genes (*e*.*g*., *heme* *oxygenase-1* (*HO-1*), Nicotinamide Adenine Dinucleotide (Phosphate) (reduced) (*NAD(P)H*) *quinone oxidoreductase-1* (*NQO1*), *catalytic subunit of glutamate*–*cysteine* (*GCLC*)). This review synthesized evidence on the mechanism through which Nrf2 modulation stabilizes atherosclerotic plaques by mitigating oxidative stress and inflammation within the vascular wall. Furthermore, we explore the cell-type-specific effects of these findings within the complex plaque microenvironment and discuss any unresolved questions, including the therapeutic potential and pharmacokinetic challenges of combining statins with direct Nrf2 activators. Thus, by extending beyond a descriptive summary, this review provides a mechanistic integration of the statin–Nrf2 axis and identifies key frontiers for future research, emphasizing the need to harness these pleiotropic effects for improved cardiovascular outcomes.

## 1. Introduction 

Dyslipidemia and the associated sequelae remain the main accused agent in cardiovascular diseases (CVDs), where dyslipidemia plays a role in the blood vessels and results in impairment and damage to the vascular tree, including the coronary arteries. Dyslipidemia, characterized by abnormal blood lipid levels, is a significant risk factor for CVDs; a condition characterized by elevated levels of total cholesterol, low-density lipoprotein (LDL) cholesterol, and triglycerides, as well as low levels of high-density lipoprotein (HDL) cholesterol [1, 2, 3, 4]. The relationship between dyslipidemia and CVDs is well established, with numerous studies demonstrating that dyslipidemia significantly increases the risk of developing conditions such as atherosclerosis, coronary artery disease, myocardial infarction, and stroke [5, 6, 7, 8, 9]. Therefore, addressing dyslipidemia through lifestyle changes and pharmacological interventions is essential to mitigate cardiovascular risk and improve patient outcomes [5, 6, 8].

Notably, statins both lower cholesterol and exhibit antioxidant and anti-inflammatory properties. Moreover, statins can increase the DNA-binding activity of nuclear factor erythroid 2-related factor 2 (Nrf2) and induce the expression of target genes, such as *heme oxygenase-1* (*HO-1*) [10]. Similarly, simvastatin reduces reactive oxygen species (ROS) levels by activating Nrf2 [11]. Furthermore, statins have been shown to modulate Apolipoprotein A1 (*ApoA1*) mRNA expression [12]. Otherwise, articular fat pad metabolism is known to be locally controlled, although correlated with, but independent of, subcutaneous white adipose tissue homeostasis in the body [13]. Meanwhile, statins are effective in both the primary and secondary prevention of cardiovascular events, and have been shown to significantly reduce cardiovascular morbidity and mortality [14, 15, 16]. Clinical trials have shown that statins can reduce the risk of coronary disease, stroke, and heart attack by lowering LDL-C levels and improving vascular health [17, 18].

Additionally, statins contribute to plaque stabilization by reducing macrophage infiltration and lipid deposition, increasing collagen content, and decreasing matrix metalloproteinase (MMP) activity. These effects are mediated by Nrf2 modulation, which strengthens the fibrous cap and reduces the volume of the necrotic core [14, 15, 19]. Therefore, statins reduce plaque vulnerability to rupturing by activating Nrf2, whose antioxidant and anti-inflammatory actions contribute to plaque instability [20], which is crucial for preventing cardiovascular events.

## 2. The Potential Molecular Mechanism of Statins in the Homeostasis of Lipid Metabolism 

Statins play a crucial role in regulating lipid homeostasis in the organism. However, the molecular mechanism through which statins maintain lipid homeostasis and reduce the risk of cardiovascular events remains unclear. Current knowledge suggests that statins block the enzyme responsible for the synthesis of LDL cholesterol in the liver, 3-hydroxy-3-methylglutaryl coenzyme A (HMG-CoA) reductase (HMGCR) [21]. Furthermore, studies over recent decades have suggested that statins have an anti-inflammatory effect, as evidenced by reduced inflammation in atherosclerotic plaques and by the ability of statins to stabilize plaques against rupture.

Statins primarily target the mevalonate pathway by inhibiting HMGCR, a crucial enzyme in cholesterol biosynthesis [22, 23, 24]. Consequently, HMGCR inhibition reduces cholesterol synthesis and upregulates LDL receptors, thereby enhancing LDL clearance from the bloodstream [22, 23, 24]. Moreover, HMGCR inhibition affects the synthesis of nonsteroidal isoprenoids, which are important for intracellular signaling pathways [25].

Statins modulate the expression of specific microRNAs (miRNAs) that regulate genes involved in lipid metabolism. For example, atorvastatin upregulates miR-129, miR-143, miR-205, miR-381, and miR-495, while downregulating miR-29b and miR-33a, which affects genes involved in lipogenesis and lipid metabolism [26]. Moreover, statins influence genes involved in unsaturated fatty acid metabolism (*e*.*g*., *stearoyl-CoA desaturase*) and cholesterol biosynthesis (*e*.*g*., *HMGCR*), which are associated with lipid droplet formation in cells [27]. Furthermore, statins inhibit protein prenylation, a process dependent on the mevalonate pathway, thereby affecting membrane targeting and the activity of small GTPases, such as those in the Rab and Rho families [28, 29]. This inhibition affects various cellular processes, including antigen processing and presentation [28]. Meanwhile, statins also modulate AMP-activated protein kinase (AMPK) activity, which regulates lipid metabolism through acetyl-CoA carboxylase (ACC) and fatty acid synthase (FAS) [30]. This pathway is crucial to maintaining cellular energy balance and lipid synthesis. Additionally, statins affect genes involved in Randomized Controlled Trial (RCT), including the cholesterol efflux from peripheral tissues, HDL metabolism, and liver internalization [12]. This modulation is complex, involving tissue-specific effects and miRNA regulation [12]. Statins exhibit anti-inflammatory properties by reducing the synthesis of inflammatory cytokines and oxidative stress markers [31, 32, 33, 34, 35]. Statins also improve endothelial function by increasing nitric oxide (NO) levels and reducing oxidative stress [34, 36].

Despite inhibiting cholesterol synthesis, some studies have shown that statins can paradoxically increase cholesterol synthesis in the liver, which is then compensated for by enhanced cholesterol excretion via bile or urine [37]. This mechanism helps to maintain lower plasma cholesterol levels (Table 1).

**Table 1.** **The mechanism for the statin-mediated effects on the organism**.

Effect	Mechanism
Inhibition of HMG-CoA reductase	Competitive inhibition of the rate-limiting enzyme in cholesterol synthesis
Increased LDL receptor expression	Enhanced clearance of LDL cholesterol from the bloodstream
Inhibition of isoprenoid synthesis	Affects cell signaling pathways involving Ras and Rho GTPases
Anti-inflammatory and antioxidative effects	Reduction in inflammatory cytokine and oxidative stress markers
Increased cholesterol excretion	Enhanced excretion through bile or feces to maintain lower plasma cholesterol levels

HMG-CoA, 3-hydroxy-3-methylglutaryl coenzyme A; LDL, low-density lipoprotein.

## 3. Statins as Modulators of Inflammation Transcription Factors 

Statins can alter gene expression through epigenetic mechanisms, such as influencing DNA methylation, histone acetylation, and miRNA regulation, which explains the associated wide-ranging (pleiotropic) effects [38]. Nrf2 is a key transcription factor that regulates the expression of a wide array of genes involved in cellular defense mechanisms, particularly against oxidative stress. The primary target genes regulated by Nrf2 include antioxidant and detoxification genes, such as *HO-1*, *NAD(P)H quinone oxidoreductase-1* (*NQO1*), *catalytic subunit of glutamate*–*cysteine* (*GCLC*), *glutamate*–*cysteine ligase modulatory subunit* (*GCLM*), *reductase of thioredoxin 1* (*TXNRD1*), and *sulfiredoxin 1* (*SRXN1*) [39, 40, 41]. Meanwhile, the cytoprotective genes include *ferritin light chain* (*FTL*) and *Notch1* [42, 43], while the metabolic and stress response genes include *uncoupling protein 1* (*UCP1*) and *myosin light chain kinase* (*MYLK*) [44, 45]. The regulatory and feedback mechanisms gene includes *BTB* and *CNC homology 1* (*Bach1*) [46, 47].

Nrf2 binds to *antioxidant response elements* (AREs) in the promoter regions of target genes to initiate transcription and forms complexes with small Maf proteins and other cofactors such as CREB-binding protein (CBP)/p300 to enhance gene transcription [43, 48]. Nrf2 regulates *Bach1* expression, which, in turn, can repress Nrf2 activity, thereby creating a feedback loop that fine-tunes the response to oxidative stress [46, 47].

The results showed that pravastatin and a cholesterol sequestrating agent, methyl-β-cyclodextrin (MβCD), can affect mRNA expression in the breast cancer cell line M. D. Anderson–Metastatic Breast 231 (MDA-MB-231) and the lung carcinoma cell line Calu-1, as compared using microarray techniques.

Treatment has been observed to cause a general downregulation not only in signal transduction, including cancer pathway proteins, but also in apoptosis and chemokine pathways, with statins affecting 35 genes by at least twofold in MDA-MB-231 cells and >300 genes in Calu-1 cells.

Furthermore, pravastatin has been shown to increase *caveolin-1* (*CAV1)* expression, decrease caveolae density, and not affect overall raft density [49]. This demonstrates the role of statins in the pathogenesis of various pathologies, including tumorigenesis. Moreover, statins influence various cellular functions, including cell migration, proliferation, and apoptosis, by inhibiting Rho GTPases [31, 50, 51]. Meanwhile, inhibition of farnesyl pyrophosphate and geranylgeranyl pyrophosphate synthesis affects the post-translational modification of proteins also involved in cell proliferation, differentiation, and apoptosis [31, 50, 51, 52].

## 4. Nrf2 is the Central Key Factor in Statin-Mediated Therapeutic Effects

Statins, such as fluvastatin and simvastatin, have been shown to activate Nrf2 through the PI3K/Akt pathway [11]. This activation leads to nuclear translocation of Nrf2, which binds to AREs and promotes transcription of antioxidant-related genes [11, 53, 54, 55]. Thus, inhibiting this pathway reduces statin-induced Nrf2 upregulation and the associated target genes, indicating a critical role for Nrf2 in the process [11, 54, 55]. Notably, the activation of Nrf2 by statins is mevalonate-dependent but cholesterol-independent, suggesting that the effects are mediated by intermediates in the mevalonate pathway rather than cholesterol [53]. Statins may also modulate the activity of Nrf2 negative regulators. Nrf2 activity is negatively regulated by several proteins, including activating transcription factor 3 (ATF3) and Bach1. These proteins can suppress Nrf2-mediated gene expression, thus regulating the cellular response to oxidative stress [46, 56], although this aspect requires further investigation.

Statins increase the expression of Nrf2 target genes, including *HO-1*, *NQO1*, and *GCLM*, which play crucial roles in cellular defense against oxidative stress [40, 53, 54]. The Nrf2/HO-1 signaling axis also plays an important role in cellular responses to oxidative stress [57]. Moreover, the Nrf2/HO-1 signaling axis regulates calcium ion levels, mitochondrial oxidative stress, and multiple cell death pathways, including ferroptosis, pyroptosis, apoptosis, and alkaliptosis, as well as processes such as autophagy and clockophagy, thereby effectively governing anti-inflammatory and antioxidant effects [57]. Upregulation of antioxidant genes by statins contributes to cytoprotective effects. For example, fluvastatin has been shown to protect vascular smooth muscle cells from oxidative damage by increasing the expression of Nrf2-related antioxidant genes, heme oxygenase-1, NAD(P)H quinone oxidoreductase-1, and glutamate–cysteine ligase modifier subunits genes through Nrf2 activation [53]. The use of PI3K/Akt inhibitors, such as LY294002, has been shown to suppress statin-induced Nrf2 activation and antioxidant enzyme expression, confirming the involvement of the PI3K/Akt pathway in this process [11, 53, 54]. In addition to the PI3K/Akt pathway, the ERK pathway similarly plays a role in statin-induced Nrf2 activation; thus, inhibiting ERK signaling likewise reduces Nrf2 activation and the expression of antioxidant enzymes [58].

Recent findings in human cardiomyocytes (HCMs) and murine skeletal muscle cells showed that atorvastatin induces mitochondria-dependent ferroptosis by modulating the Nrf2-xCT/GPx4 axis, which accounts for the undesirable muscular effect [59]. The study examined the viability of HCM and C2C12 cells and suggested that the underlying molecular mechanism of the maintained effects of atorvastatin occurs in the mitochondria in a dose-dependent manner and is associated with significant increases in lipid peroxidation, intracellular iron ions, and ROS [59]. Furthermore, the study demonstrated that ferroptosis is involved in the pathophysiology of statin-associated myopathy [59]. Meanwhile, the observed downregulation of ferroptosis in human myocardiocytes after glutathione (GSH) depletion and the reduction of Nrf2, glutathione peroxidase 4 (GPx4), and glutamate–cysteine antiporter xCT, the main component of which is SLC7A11, can serve as a novel therapeutic target in the management of atorvastatin-associated myopathy [59].

## 5. Integrated Mechanistic Framework: From Nrf2 Activation to Plaque Stabilization

Statins exert pleiotropic effects, largely by activating Nrf2, a central regulator of cellular redox homeostasis and inflammatory responses. The mechanistic cascade begins with statin-induced inhibition of HMG-CoA reductase, which depletes the mevalonate pathway intermediates, particularly geranylgeranyl pyrophosphate (GGPP) [60]. This depletion activates key kinase pathways, including PI3K/Akt and ERK, which phosphorylate Nrf2 and facilitate the dissociation of Nrf2 from the cytosolic repressor Keap1. Once liberated, Nrf2 translocates to the nucleus and binds to AREs in the promoter regions of cytoprotective genes [61].

Among the most critical targets of Nrf2 is HO-1, whose induction plays a pivotal role in mitigating oxidative stress. HO-1 catalyzes the degradation of pro-oxidant heme into biliverdin, carbon monoxide, and free iron, each of which contributes to antioxidant and anti-inflammatory effects [62]. Biliverdin is subsequently converted to bilirubin, a potent ROS scavenger, while carbon monoxide exerts vasoprotective and antiapoptotic effects. Concurrently, Nrf2 activation upregulates other key enzymes, such as NQO1, and subunits of glutamate–cysteine ligase (GCLC/GCLM), thereby enhancing cellular glutathione biosynthesis and redox buffering capacity [63].

**Fig. 1.**
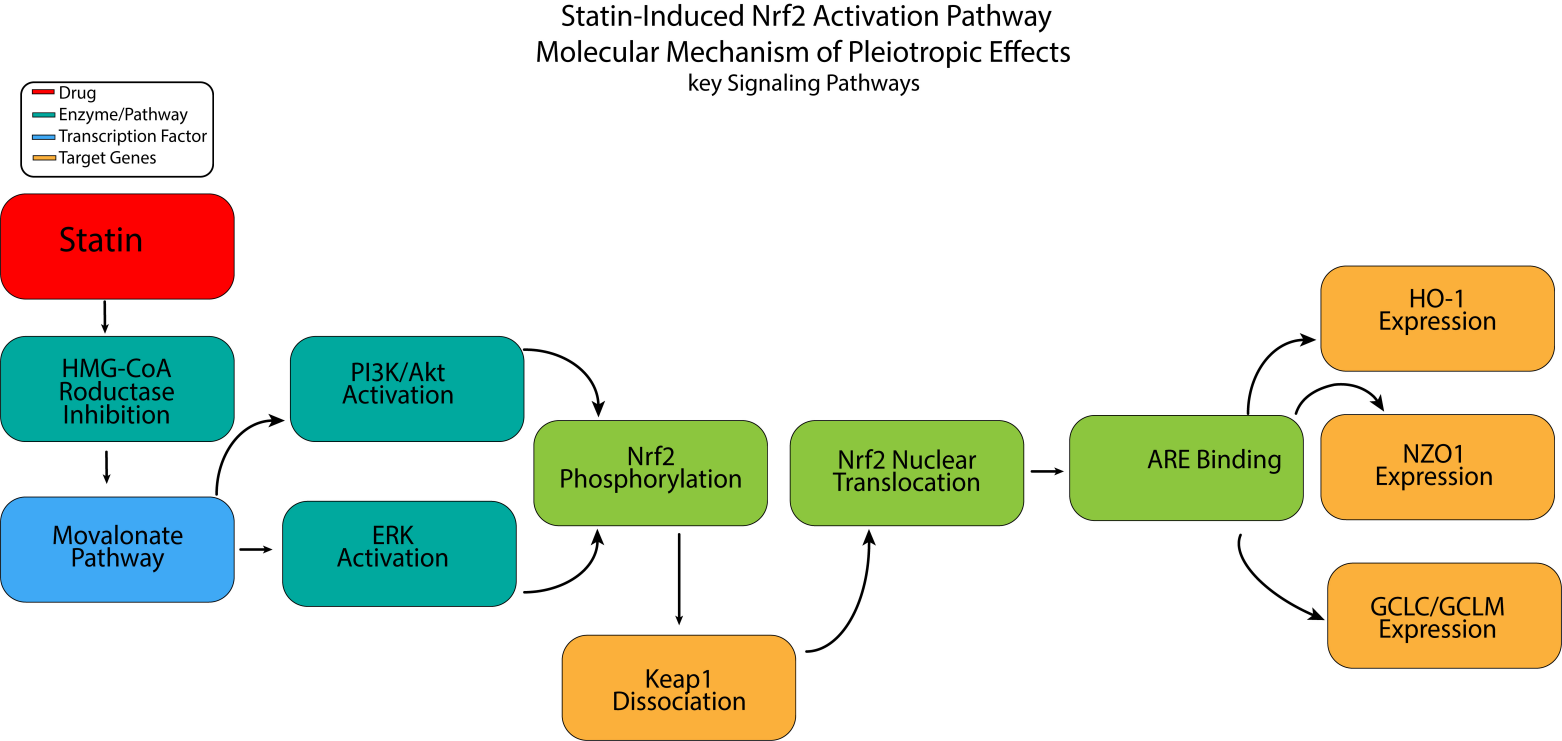
**Mechanism of the Statin–Nrf2 signaling pathway**. Nrf2, nuclear factor erythroid 2-related factor 2; ARE, antioxidant response element; HO-1, heme oxygenase-1; NQO1, NAD(P)H quinone oxidoreductase-1; GCLC, catalytic subunit of glutamate–cysteine ligase; GCLM, glutamate–cysteine ligase modulatory subunit. Figure created through Visual Studio 2026 using Python code.

This coordinated gene expression profile directly counteracts several pathological processes in atherosclerosis. Thus, reducing oxidative stress by scavenging ROS and inhibiting lipid peroxidation limits the oxidative modification of LDL, a key trigger of foam cell formation. Furthermore, HO-1 and other Nrf2 targets suppress inflammation by inhibiting NF-κB activation, thereby reducing the production of proinflammatory cytokines and chemokines that recruit monocytes to the plaque [64]. Concurrently, Nrf2 activation enhances endothelial protection by increasing NO bioavailability and reducing adhesion molecule expression, thereby improving endothelial function and attenuating leukocyte adhesion. Consequently, through these combined antioxidant, anti-inflammatory, and vasoprotective effects, statins promote a stable plaque phenotype characterized by reduced macrophage infiltration, increased collagen content, and a smaller necrotic core (Fig. 1).

## 6. Nrf2 as a Novel Therapeutic Target in the Management of Hyperinflammation and Dyslipidemia

**Fig. 2.**
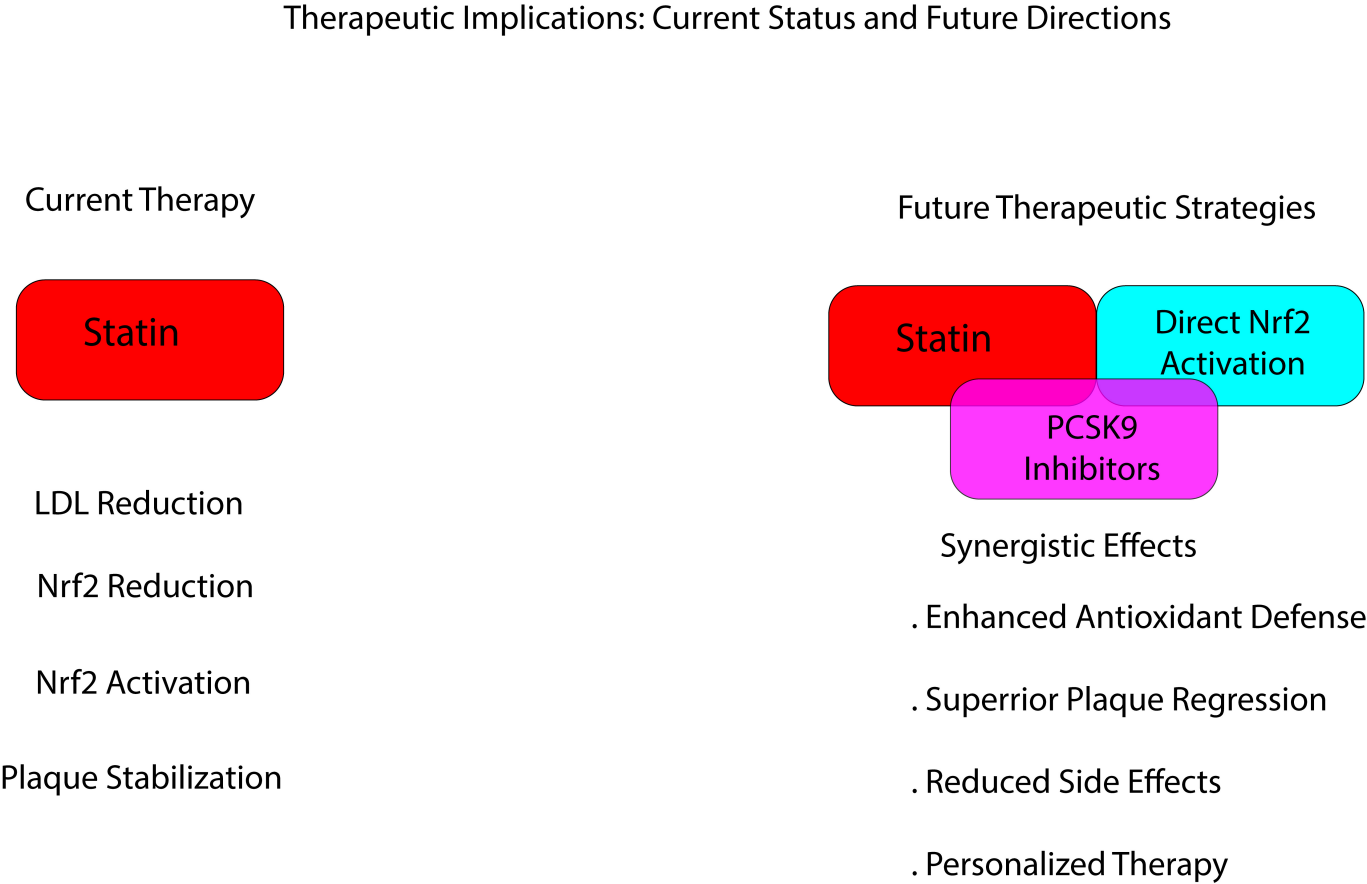
**Therapeutic implications of Nrf2 as a novel therapeutic target in the management of hyperinflammation and dyslipidemia**. PCSK9, proprotein convertase subtilisin/kexin type 9. Figure created through Visual Studio 2026 using Python code.

The ability of statins to activate Nrf2 and upregulate the associated target genes suggests a potential therapeutic role in conditions characterized by oxidative stress and inflammation [40, 65]. Indeed, the dysregulation of Nrf2 and the related target genes, such as *Bach1*, has been implicated in conditions such as Parkinson’s disease [46]. Nrf2 target genes play a significant role in diseases characterized by oxidative stress, including CVDs and diabetes (Fig. 2) [66]. Thus, the modulation of the Nrf2/HO-1 pathway by statins has potential therapeutic implications for various diseases characterized by oxidative stress, including CVDs, neurodegenerative disorders, and inflammatory conditions [10, 40, 53, 54]. Statins activate Nrf2 and enhance the associated antioxidant protective effects, improving plaque stability and reducing the risk of cardiovascular events. This highlights the importance of considering both lipid-lowering and pleiotropic effects of statins in the management of atherosclerosis [67, 68, 69]. Indeed, statins can help stabilize atherosclerotic plaques by modulating Nrf2 expression and activity through reducing oxidative stress and inflammation and promoting antioxidant defenses within the plaque environment [67, 68, 70, 71, 72, 73]. Meanwhile, Nrf2 modulates the expression of antioxidant genes, scavenger receptors, and cholesterol efflux transporters, thereby contributing to foam cell development and plaque formation [74]. The ApoA1/Narf2/HO-1 axis may also represent a novel therapeutic target. Furthermore, enhancing Nrf2 activity with statin medications may restore cholesterol efflux, reduce ApoA1 deposition, and alleviate knee osteoarthritis (OA).

Statins inhibit the expression of inflammatory cytokines [75], C-reactive protein (CRP) [76, 77], interleukins, tumor necrosis factor (TNF), and Matrix metallopeptidase (MMP) *in vivo*.

## 7. Current Advances and Future Perspectives 

Statins can improve endothelial function, stabilize atherosclerotic plaques, decrease oxidative stress and inflammation, and inhibit thrombogenic response [78, 79, 80, 81, 82, 83]. These benefits contribute to cardiovascular protection beyond just lowering LDL cholesterol [81, 83]. Additionally, statins exhibit anti-inflammatory and antioxidant effects, mediated by inhibiting isoprenoid synthesis and thereby modulating intracellular signaling molecules such as Rho, Ras, and Rac [11, 78, 79, 80, 82, 83, 84, 85]. Indeed, statins have shown potential benefits in the treatment of conditions unrelated to cholesterol levels, such as chronic heart failure, rheumatoid arthritis, multiple sclerosis, sepsis, cancer, and dementia [81, 82, 86, 87, 88, 89, 90, 91, 92, 93, 94, 95, 96, 97, 98]. Moreover, statins may protect against ischemic injury and have cytoprotective actions, including promoting angiogenesis and modulating inflammatory responses [78, 79, 83, 99]. However, despite promising advances, several challenges remain, including the fact that increased blood glucose levels have been associated with a higher risk of diabetes in patients taking statins [84, 100, 101]. Thus, while the evidence supporting the role of statins in activating Nrf2 and promoting antioxidant and anti-inflammatory responses is compelling, several critical questions and limitations in the current research landscape remain unresolved. Therefore, recognizing these gaps is essential for guiding future scientific inquiries and translating these mechanisms into improved clinical strategies [10, 102].

A significant, yet underexplored, area is the potential for pharmacokinetic interactions between statins and novel Nrf2-targeting agents. Many statins, particularly simvastatin, lovastatin, and atorvastatin, are metabolized by the cytochrome P450 3A4 (CYP3A4) enzyme [102]. Hence, concomitant administration of potent Nrf2 activators, such as bardoxolone methyl, could potentially modulate the activity of these metabolic enzymes, thus altering statin plasma concentrations. This raises crucial questions about the risk of statin-related adverse effects, for example, myopathy, or, conversely, reduced efficacy. Future studies must systematically evaluate these drug–drug interactions in both preclinical models and clinical settings to establish safe and effective dosing regimens for combination therapies [103].

Atherosclerotic plaques represent a complex ecosystem comprising endothelial cells, macrophages, and vascular smooth muscle cells (VSMCs). Current evidence often generalizes the statin–Nrf2 mechanism across these cell types [104, 105]. However, it is highly plausible that the response to Nrf2 activation is cell-specific. For example, while Nrf2 activation in macrophages may enhance antioxidant defenses and reduce foam cell formation, the associated effects on VSMC proliferation and phenotype switching could have dual implications for plaque stability. Therefore, future research should employ cell-specific knockout models (for example, using Cre-Lox technology) to delineate the precise contribution of Nrf2 in each cellular component of the plaque [74]. This will clarify whether global Nrf2 activation is uniformly beneficial or if a more targeted, cell-specific approach is warranted [74].

**Fig. 3.**
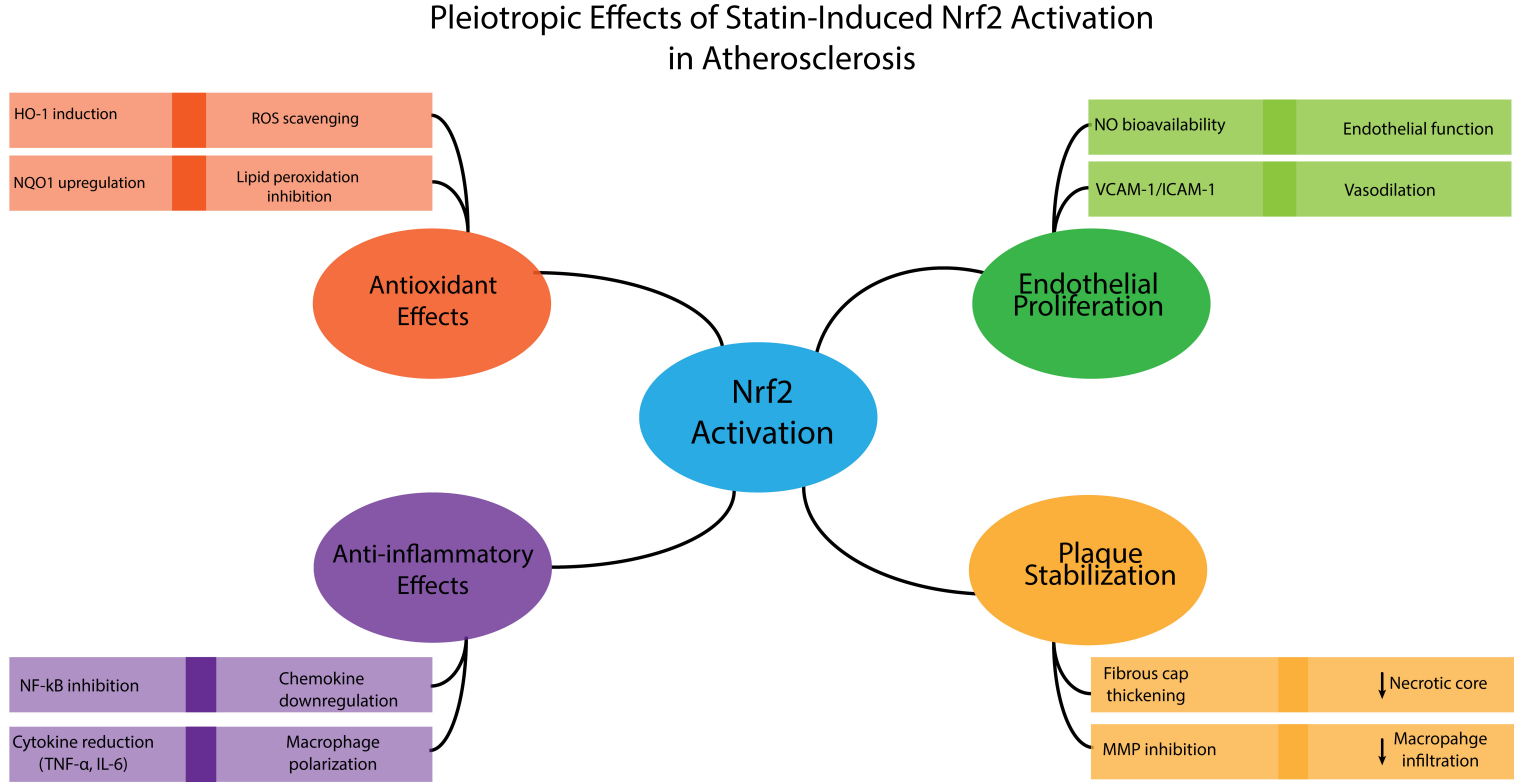
**Pleiotropic effects of Nrf2 activation in atherosclerosis**. ROS, reactive oxygen species; NO, nitric oxide; MMP, matrix metalloproteinase; VCAM-1, Vascular Cell Adhesion Molecule-1; ICAM-1, Intercellular Adhesion Molecule-1. The arrow means reduction or decrease. Figure created through Visual Studio 2026 using Python code.

In addition to statins, several direct Nrf2 activators, such as bardoxolone methyl and sulforaphane, are being investigated for various conditions. The potential synergistic effects of combining these agents with statins represent a promising but largely unexplored therapeutic frontier [106]. Such combinations could, in principle, enable lower doses of each drug, thereby mitigating side effects while achieving superior anti-atherosclerotic efficacy through complementary mechanisms (*e*.*g*., intense antioxidant induction coupled with potent lipid-lowering). Preclinical studies in animal models of atherosclerosis are urgently needed to test this interaction and evaluate outcomes such as plaque burden, composition, and stability (Fig. 3) [106].

## 8. Discussion 

Statins modulate lipid metabolism through a dual approach: (1) direct inhibition of cholesterol synthesis and upregulation of Low-Density Lipoprotein Receptor (LDLR), and (2) pleiotropic effects mediated by isoprenoid depletion [78]. These mechanisms collectively reduce the burden and cardiovascular risk. Ongoing research on complementary therapies (*e*.*g*., Proprotein Convertase Subtilisin/Kexin Type 9 (PCSK9) inhibitors) continues to refine strategies for optimizing cholesterol homeostasis [107, 108].

Certain populations, such as people with diabetes, are at a higher risk of dyslipidemia and the related cardiovascular consequences. Diabetic dyslipidemia, characterized by high triglyceride and low HDL levels, significantly increases the risk of coronary heart disease and other cardiovascular events [109, 110, 111]. Furthermore, secondary causes of dyslipidemia, such as hypothyroidism and obesity, should be identified and managed to effectively control lipid levels and reduce cardiovascular risk[112] (Table 2) (Ref. [67, 68, 70, 71, 72, 74, 113, 114, 115, 116]).

**Table 2.** **Mechanisms of Nrf2 therapeutic potential in atherosclerotic plaques**.

Aspect	Nrf2	Statins	Transcription factors
Role in atherosclerosis	Regulates oxidative stress, inflammation, autophagy, and senescence [70, 71, 72, 74]	Reduces lipid levels, oxidative stress, and inflammation through Nrf2 activation [67, 68, 113]	Interacts with the NF-κB, JAK/STAT, PI3K/Akt pathways [114, 115]
Mechanisms	Promotes autophagy, inhibits senescence, modulates lipid metabolism [74]	Activates Nrf2 via the PI3K/Akt pathway, reduces cytokine secretion [67, 68]	Regulates endothelial function, vascular smooth muscle cell behavior [116]
Therapeutic potential	Target for antioxidant and anti-inflammatory therapies [70, 71, 74]	Potential to prevent atherosclerosis and abdominal aortic aneurysms [113]	Modulates inflammatory and oxidative stress responses [114, 115]

## 9. Conclusion 

Statins activate the Nrf2 transcription factor, leading to the upregulation of several antioxidant and cytoprotective genes, including *HO-1*, *NQO1*, *GCLC*, and *GCLM*. These downstream targets play a significant role in mitigating oxidative stress and inflammation, thus contributing to the pleiotropic effects of statins [53, 54, 117].

## Availability of Data and Materials

Not applicable.
